# Target degradation specificity of phytoplasma effector phyllogen is regulated by the recruitment of host proteasome shuttle protein

**DOI:** 10.1111/mpp.13410

**Published:** 2023-12-17

**Authors:** Masato Suzuki, Yugo Kitazawa, Nozomu Iwabuchi, Kensaku Maejima, Juri Matsuyama, Oki Matsumoto, Kenro Oshima, Shigetou Namba, Yasuyuki Yamaji

**Affiliations:** ^1^ Department of Agricultural and Environmental Biology, Graduate School of Agricultural and Life Sciences The University of Tokyo Tokyo Japan; ^2^ Faculty of Bioscience, Hosei University Tokyo Japan

**Keywords:** effector target specificity, MADS‐box transcription factor, phyllogen, phytoplasma, protein interaction, RAD23

## Abstract

Phytoplasmas infect a wide variety of plants and can cause distinctive symptoms including the conversion of floral organs into leaf‐like organs, known as phyllody. Phyllody is induced by an effector protein family called phyllogens, which interact with floral MADS‐box transcription factors (MTFs) responsible for determining the identity of floral organs. The MTF/phyllogen complex then interacts with the proteasomal shuttle protein RADIATION SENSITIVE23 (RAD23), which facilitates delivery of the MTF/phyllogen complex to the host proteasome for MTF degradation. Previous studies have indicated that the MTF degradation specificity of phyllogens is determined by their ability to bind to MTFs. However, in the present study, we discovered a novel mechanism determining the degradation specificity through detailed functional analyses of a phyllogen homologue of rice yellow dwarf phytoplasma (PHYL_RYD_). PHYL_RYD_ degraded a narrower range of floral MTFs than other phyllody‐inducing phyllogens, resulting in compromised phyllody phenotypes in plants. Interestingly, PHYL_RYD_ was able to bind to some floral MTFs that PHYL_RYD_ was unable to efficiently degrade. However, the complex of PHYL_RYD_ and the non‐degradable MTF could not interact with RAD23. These results indicate that the MTF degradation specificity of PHYL_RYD_ is correlated with the ability to form the MTF/PHYL_RYD_/RAD23 ternary complex, rather than the ability to bind to MTF. This study elucidated that phyllogen target specificity is regulated by both the MTF‐binding ability and RAD23 recruitment ability of the MTF/phyllogen complex.

## Graphical abstract

Phyllogen‐mediated degradation specificity for MADS transcription factors (MTFs) is controlled by two steps: MTF‐binding affinity and recruitment of proteasome shuttle protein (this study, red arrow).

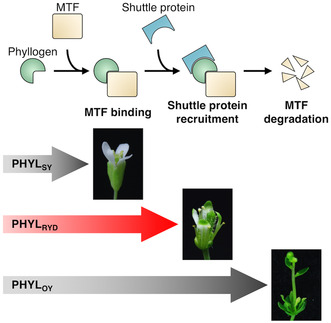

## 1 INTRODUCTION

Plant pathogens establish colonization on their host plants by secreting dozens of effector proteins to manipulate host developmental processes and suppress immune responses (Hogenhout et al., 2009). Functional analysis of effector proteins provides valuable information to understand the infection mechanisms of plant pathogens. Effector genes are usually exposed to selective pressures from the host plant, located in highly variable regions on pathogen genomes and undergo genetic mutations that result in differences in activity and functional diversification among homologous effectors (Raffaele & Kamoun, 2012; Upson et al., 2018). For example, Bentham et al. (2021) reported that APikL2, an effector conserved among blast fungus isolates from different grass hosts, has different amino acid polymorphisms in accordance with the host plant species, one of which broadens the binding specificity of APikL2 to its target host proteins. Thus, studying the functional variation within an effector family provides valuable insights into the molecular mechanisms of effector target specificity and co‐evolution with host plant species, contributing to a better understanding of the roles of the effector family in pathogenicity.

Phytoplasmas (“*Candidatus* Phytoplasma” spp.) are a group of plant‐pathogenic bacteria in class Mollicutes that are associated with various diseases in more than 1000 plant species (Marcone, 2014). Phytoplasma infection causes unique developmental abnormalities such as witches' broom (proliferation of tiny shoots with small leaves) and phyllody (transformation of floral organs into leaf‐like structures); several causal effectors have been identified to date (Huang et al., 2021; Namba, 2019; Oshima et al., 2023). Phyllogens, a phyllody‐inducing gene family, are among of the most intensively studied effectors conserved among phytoplasma species (Iwabuchi et al., 2020; MacLean et al., 2011; Maejima, Iwai, et al., 2014). Phyllogens induce phyllody in various plant species by targeting plant‐conserved MADS‐box transcription factors (MTFs) involved in floral development (Kitazawa et al., 2017). Floral MTFs are classified into classes A–E and cooperatively regulate floral organ differentiation (Kaufmann et al., 2005; Theißen & Saedler, 2001). Phyllogens specifically interact with A‐ and E‐class floral MTFs and induce their degradation through a proteasome‐mediated pathway (MacLean et al., 2014; Maejima, Iwai, et al., 2014). Phyllogens also interact with RADIATION SENSITIVE23 (RAD23), a shuttle protein that delivers ubiquitinated proteins to the proteasome (MacLean et al., 2014). Recently, phyllogen‐mediated MTF degradation was demonstrated to occur through the following steps: the phyllogen interacts with a target MTF; the MTF/phyllogen complex binds directly to RAD23 without ubiquitin; RAD23 delivers the complex to the proteasome, resulting in ubiquitin‐independent proteasomal degradation of the target MTF (Kitazawa et al., 2022).

Several studies have reported phyllogens with impaired phyllody‐inducing activity and MTF degradation activity (Aurin et al., 2020; Iwabuchi et al., 2019, 2020; Liao et al., 2019). All of these phyllogens have defects in MTF‐binding affinity, and the residues responsible for interaction with MTFs are concentrated on one surface of the phyllogen structure, which is a putative MTF‐binding interface (Kitazawa et al., 2023). Based on these findings, together with the fact that phyllogen binds only to specific MTFs to induce their degradation (MacLean et al., 2014), the target specificity of phyllogens has been postulated to be determined by their MTF‐binding affinity.

Our previous study revealed sequence diversity among phyllogen homologues and classified the homologues into groups phyl‐A to ‐D (Iwabuchi et al., 2020). Interestingly, although the examined phyllogens in the phyl‐A, C and D groups induced similar phyllody phenotypes in plants, all phyl‐B phyllogens were found to completely lack phyllody‐inducing activity. Site‐directed mutagenesis analyses revealed that a single amino acid substitution conserved in the phyl‐B group abolishes the MTF‐binding affinity of phyllogens, resulting in defects in MTF degradation activity.

The “*Candidatus* Phytoplasma oryzae” rice yellow dwarf strain (RYD phytoplasma) is one of the most important pathogens of rice, causing severe systemic symptoms such as leaf yellowing, severe stunting and excessive tillering (Jung et al., 2003; Maejima, Oshima, & Namba, 2014). Notably, RYD phytoplasma induces floral abnormality in rice ears, including spikelet sterility and reduced ear numbers (Komori, 1965), although phyllody symptoms have not been reported. The phyllogen from RYD phytoplasma (PHYL_RYD_) is the only full‐length phyllogen identified from cereal‐infecting phytoplasmas (Iwabuchi et al., 2020; Zhu et al., 2017). Although PHYL_RYD_ belongs to the phyl‐A group, it has multiple unique polymorphisms compared with other members in this group (Iwabuchi et al., 2020). Despite these intriguing characteristics, functional characterization of PHYL_RYD_ has not yet been performed. Therefore, in this study, we investigated the phyllody‐inducing activity and related functions of PHYL_RYD_.

## 2 RESULTS

### 2.1 Sequence‐based features of PHYL_RYD_

**FIGURE 1:**
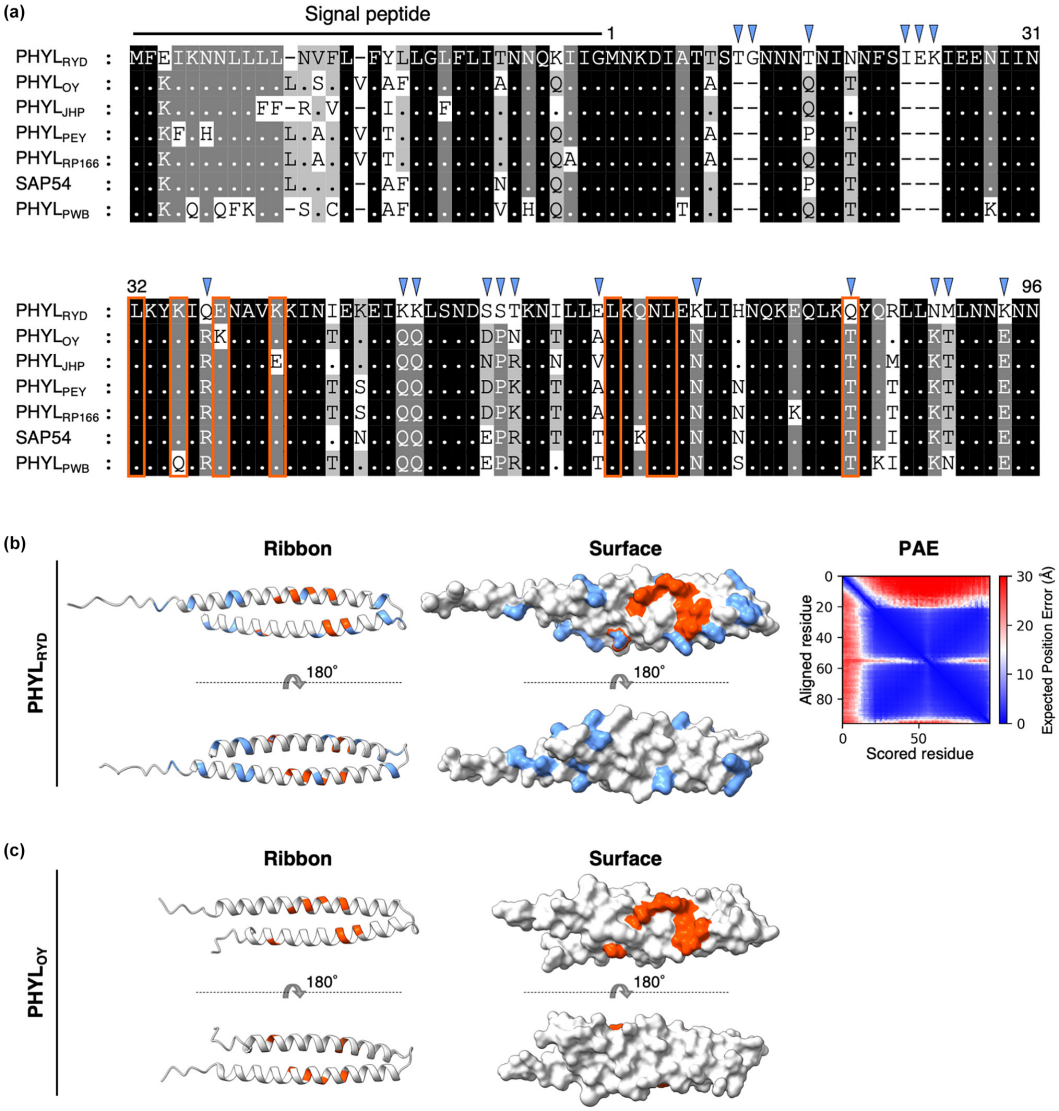
PHYL_RYD_ possesses multiple unique polymorphisms but retains the basic structure. (a) Protein sequence comparisons between PHYL_RYD_ and other phyl‐A group phyllogens with phyllody‐inducing activity. Sequences were aligned using MEGA v. 10.1.8 (Kumar et al., 2018) with the MUSCLE algorithm (Edgar, 2004). Light blue arrowheads indicate insertions and amino acid substitutions specific to PHYL_RYD_. The residues considered to constitute the putative MADS box transcription factor (MTF)‐binding interface (Kitazawa et al., 2023; Tokuda et al., 2023) are surrounded in orange. Numbers indicate the amino acid number of the putative secreted region of PHYL_RYD_. The background colour indicates the percentage of amino acid similarity: black 100%; dark grey 80%; light grey 60%. (b) The predicted three‐dimensional structure of PHYL_RYD_. The structure of the secreted region of PHYL_RYD_ was predicted using ColabFold (Mirdita et al., 2022). The PHYL_RYD_‐specific polymorphisms are shown in light blue, and the residues involved in MTF‐binding are shown in orange similarly as in (a). The predicted aligned error (PAE) is shown on the right. The predicted local distance difference test (pLDDT) score and the predicted TM (pTM) score were 85.6 and 0.69, respectively. (c) The crystal structure of PHYL_OY_ (PDB ID: 6JQA; Iwabuchi et al., 2019). PHYL_OY_ is one of the phyl‐A group phyllogens with phyllody‐inducing activity. The residues involved in MTF‐binding are shown in orange.

The phylogenetic tree of the phyl‐A group displayed a large evolutionary distance between PHYL_RYD_ and other phyllogens (Figure S1; Iwabuchi et al., 2020). Phyl‐A phyllogens other than PHYL_RYD_ share 85%–100% nucleotide (nt) and 72%–100% amino acid (aa) identity; however, PHYL_RYD_ shows 82%–88% nt and 67%–76% aa identity with other phyl‐A group phyllogens. Protein sequence alignment has revealed that the putative secreted region of PHYL_RYD_ has two short insertions and 13 unique amino acid substitutions (Figure 1a) when compared to other phyllody‐inducing phyllogens in the phyl‐A group (Iwabuchi et al., 2020; MacLean et al., 2014; Tokuda et al., 2023). However, the residues considered to constitute the putative binding interface with MTF (Kitazawa et al., 2023; Tokuda et al., 2023) are conserved, except for the glutamine at position 83 (Figure 1a). In silico structure prediction using ColabFold (Mirdita et al., 2022) suggests that despite the multiple polymorphisms, the putative secreted region of PHYL_RYD_ consists of two α‐helices, similar to other phyllogens (Figure 1b,c; Iwabuchi et al., 2019; Liao et al., 2019). The PHYL_RYD_‐specific polymorphisms were not concentrated on either the putative MTF‐binding interface (Kitazawa et al., 2023) or other specific regions (Figure 1b,c).

### 2.2 Phyllody‐inducing activity of PHYL_RYD_

**FIGURE 2:**
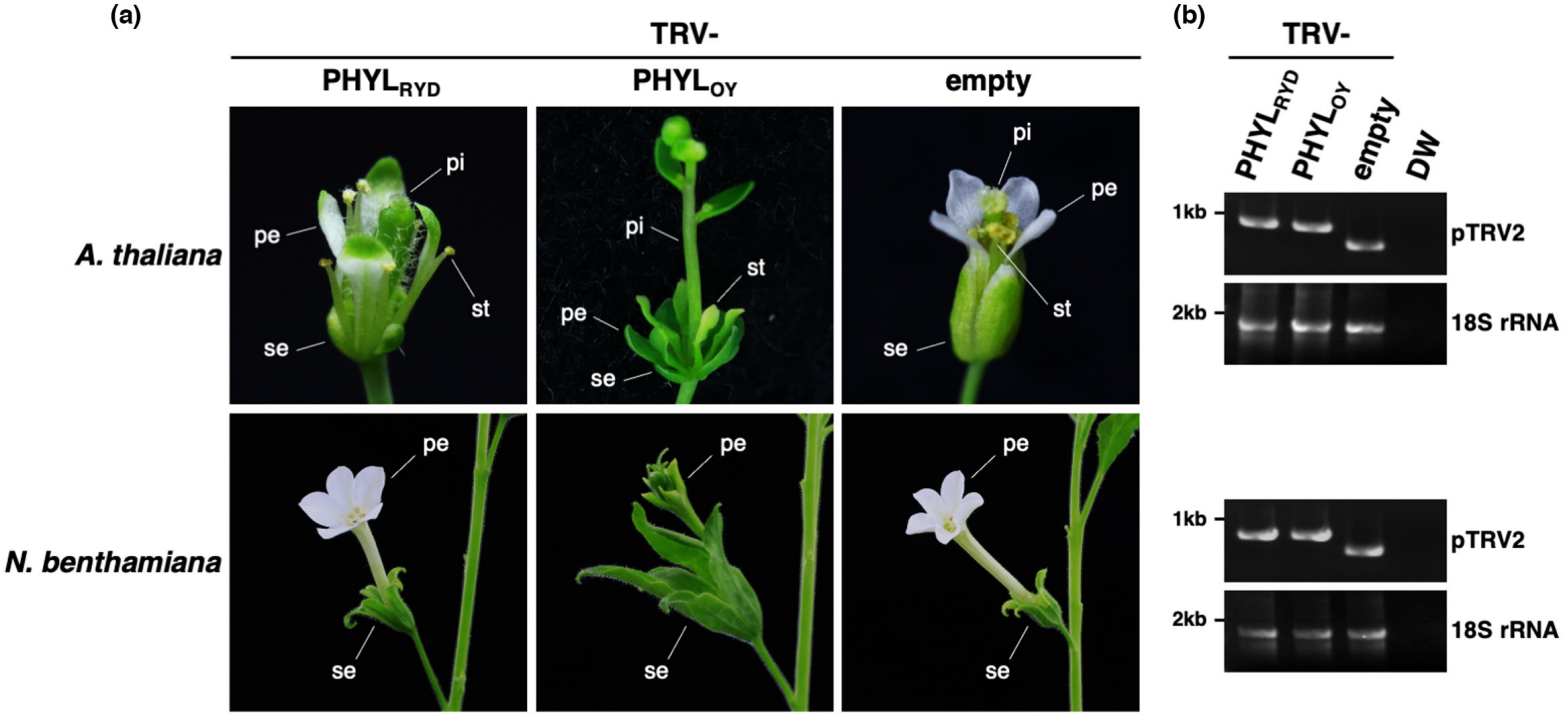
Phyllody‐inducing activity of PHYL_RYD_ is compromised. (a) Floral phenotypes of phyllogen‐expressing plants. *Arabidopsis thaliana* and *Nicotiana benthamiana* were infected with tobacco rattle virus (TRV) vector carrying either PHYL_RYD_, PHYL_OY_ or no exogenous gene (empty). Sepals, petals, stamens and pistils are indicated as se, pe, st and pi, respectively. (b) Confirmation of phyllogen expression. Total RNA was extracted from the flowers and reverse transcription (RT)‐PCR was conducted to amplify the insertion region in pTRV2. 18S ribosomal RNA (rRNA) was also amplified as an internal control. PCR using distilled water (DW) as a template was performed for a negative control.

To examine the phyllody‐inducing activity of PHYL_RYD_, it was expressed in *Arabidopsis thaliana* and *Nicotiana benthamiana* by a modified tobacco rattle virus (TRV)‐based gene expression vector (Iwabuchi et al., 2019). PHYL_OY_, a well‐studied phyl‐A group phyllogen of the “*Candidatus* Phytoplasma asteris” onion yellows strain (Kitazawa et al., 2022; Maejima, Iwai, et al., 2014), was used as a positive control. In *A. thaliana* plants, PHYL_OY_ induced phyllody and virescence of the flowers, as described previously (Iwabuchi et al., 2019); the sepals, petals and stamens were converted into green leaf‐like organs and the pistil changed into a stem‐like structure (Figure 2a, upper panels). In contrast, PHYL_RYD_‐expressing plants exhibited much less severe phenotypes than PHYL_OY_‐expressing plants; the sepals often became small, and the petals sometimes turned greenish. However, homeotic conversions of the stamens into leaf‐like organs and the pistil into the stem‐like structure were rarely observed. In *N. benthamiana*, although >95% of PHYL_OY_‐expressing flowers exhibited malformation or phyllody, >85% of PHYL_RYD_‐expressing flowers showed no abnormalities (Figure 2a, lower panels; Figure S2). The expression of PHYL_RYD_ or PHYL_OY_ in the flowers of each plant species was confirmed by reverse transcription (RT)‐PCR of the phyllogen‐insertion region in pTRV2 (Figure 2b). These results indicated that the phyllody‐inducing activity of PHYL_RYD_ was compromised in *A. thaliana* and *N. benthamiana*, albeit not completely lost.

### 2.3 MTF degradation activity of PHYL_RYD_

**FIGURE 3:**
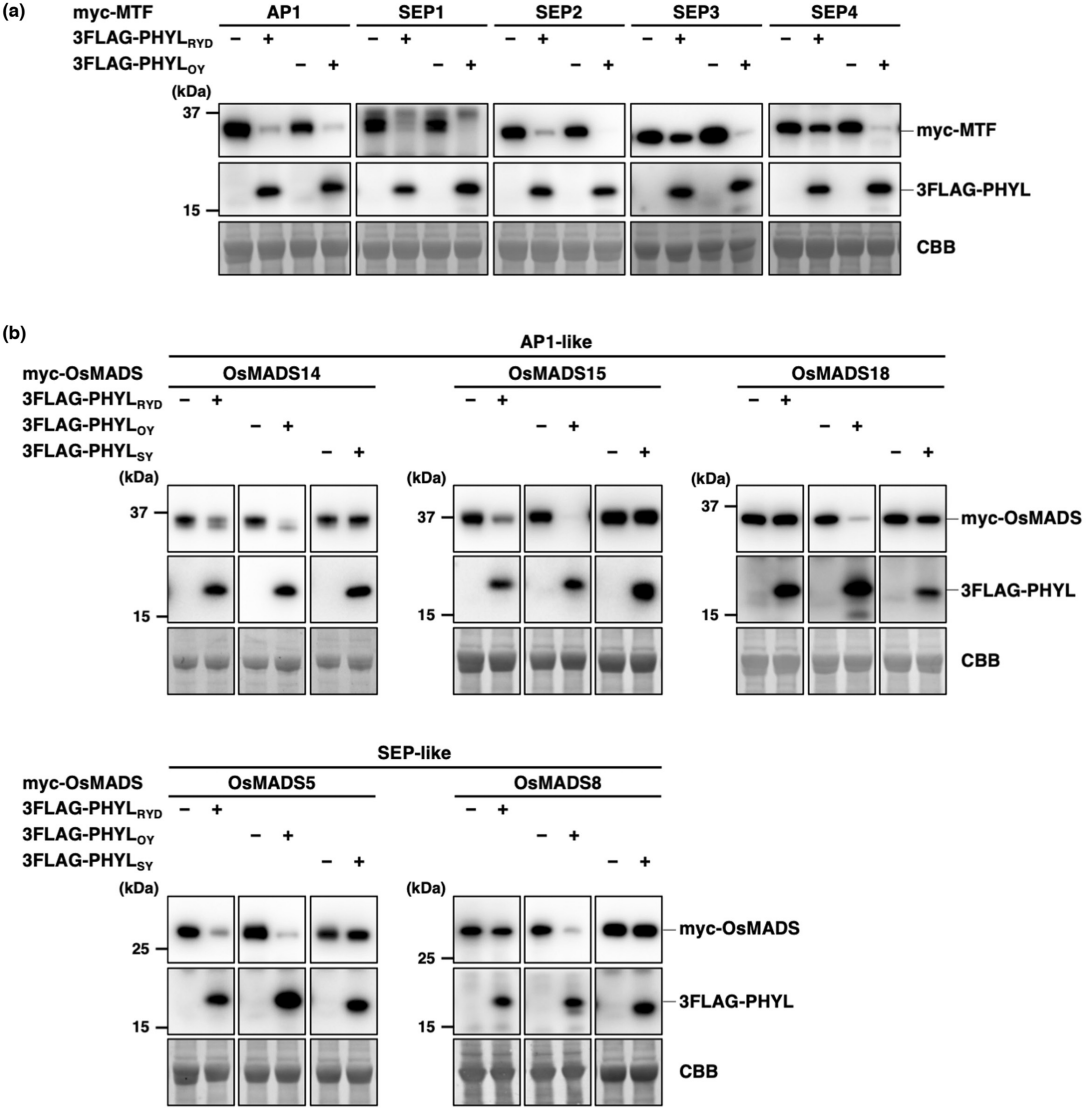
Degradation activity of PHYL_RYD_ for several MADS box transcription factors (MTFs) is reduced. (a) The degradation activity of PHYL_RYD_ for *Arabidopsis* MTFs. Each myc‐fused MTF (myc‐MTF) was transiently expressed with or without 3FLAG‐fused PHYL_RYD_ (3FLAG‐PHYL_RYD_) in *Nicotiana benthamiana* leaves by agroinfiltration. 3FLAG‐fused PHYL_OY_ (3FLAG‐PHYL_OY_) was used as a positive control. Accumulation of myc‐MTF and 3FLAG‐PHYL was evaluated by immunoblotting using anti‐myc and anti‐FLAG antibodies, respectively. Membranes stained by Coomassie brilliant blue (CBB) are shown as loading controls. (b) The degradation activity of PHYL_RYD_ for AP1‐like and SEP‐like *Oryza* MTFs. In addition to PHYL_RYD_, phyllody‐inducing phyllogen PHYL_OY_ and non‐phyllody‐inducing phyllogen PHYL_SY_ were used. The experimental conditions were as described in (a).

Phyllogens including PHYL_OY_ degrade APETALA1 (AP1) and SEPALLATA (SEP) 1–4 of *A. thaliana* (A‐ and E‐class MTFs, respectively) and defects in their MTF degradation activity cause the loss of phyllody‐inducing activity (Iwabuchi et al., 2020). To clarify the molecular mechanism of the significant attenuation of the phyllody phenotype induced by PHYL_RYD_, the MTF degradation activity of PHYL_RYD_ was compared with that of PHYL_OY_ (Figure 3a). Myc‐fused AP1 and SEP1–4 (myc‐AP1 and myc‐SEP1–4) were transiently expressed with and without 3FLAG‐fused phyllogen (3FLAG‐PHYL_RYD_ and 3FLAG‐PHYL_OY_) in *N. benthamiana* leaves, and protein accumulation was analysed by immunoblotting. Co‐expression of 3FLAG‐PHYL_RYD_ significantly decreased the accumulation of myc‐AP1, myc‐SEP1 and myc‐SEP2 to levels comparable to those of PHYL_OY_, indicating that PHYL_RYD_ has degradation activity for these MTFs (PHYL_RYD_‐degradable MTFs). In contrast, degradation activity was much lower for myc‐SEP3 and myc‐SEP4 (non‐PHYL_RYD_‐degradable MTFs) (Figure 3a).

Because the natural host plant of RYD phytoplasma is rice (*Oryza sativa*), we speculated that PHYL_RYD_ may have adapted to degrade MTFs of *O. sativa* (*Oryza* MTFs), rather than MTFs of *A. thaliana* (*Arabidopsis* MTFs). To test this possibility, the degradation activity of PHYL_RYD_ for *Oryza* MTFs belonging to the AP1‐like clade (OsMADS14, OsMADS15 and OsMADS18) and SEP‐like clade (OsMADS5 and OsMADS8) was also evaluated (Figure 3b). In addition to PHYL_RYD_ and PHYL_OY_, we used PHYL_SY_, a non‐phyllody‐inducing phyl‐B group phyllogen of the “*Candidatus* Phytoplasma fragariae” strawberry yellows strain (Iwabuchi et al., 2020). The results showed that all the tested *Oryza* MTFs were degraded by PHYL_OY_, but not at all by PHYL_SY_, which is consistent with the findings of a previous study using *Arabidopsis* MTFs (Iwabuchi et al., 2020). Compared with PHYL_OY_, degradation activity of PHYL_RYD_ was similar or slightly inferior for OsMADS14, OsMADS15, and OsMADS5, but significantly lower for OsMADS8 and OsMADS18. These results do not support the idea that PHYL_RYD_ has evolved to efficiently degrade *Oryza* MTFs. Rather, it is likely that the range of floral MTFs degraded by PHYL_RYD_ is narrower than that degraded by PHYL_OY_ for both *Arabidopsis* and *Oryza* MTFs. No clear correlation was found between the phylogenetic relationship of MTFs and degradation activity by PHYL_RYD_ (Figure S3).

### 2.4 MTF‐binding affinity of PHYL_RYD_

**FIGURE 4:**
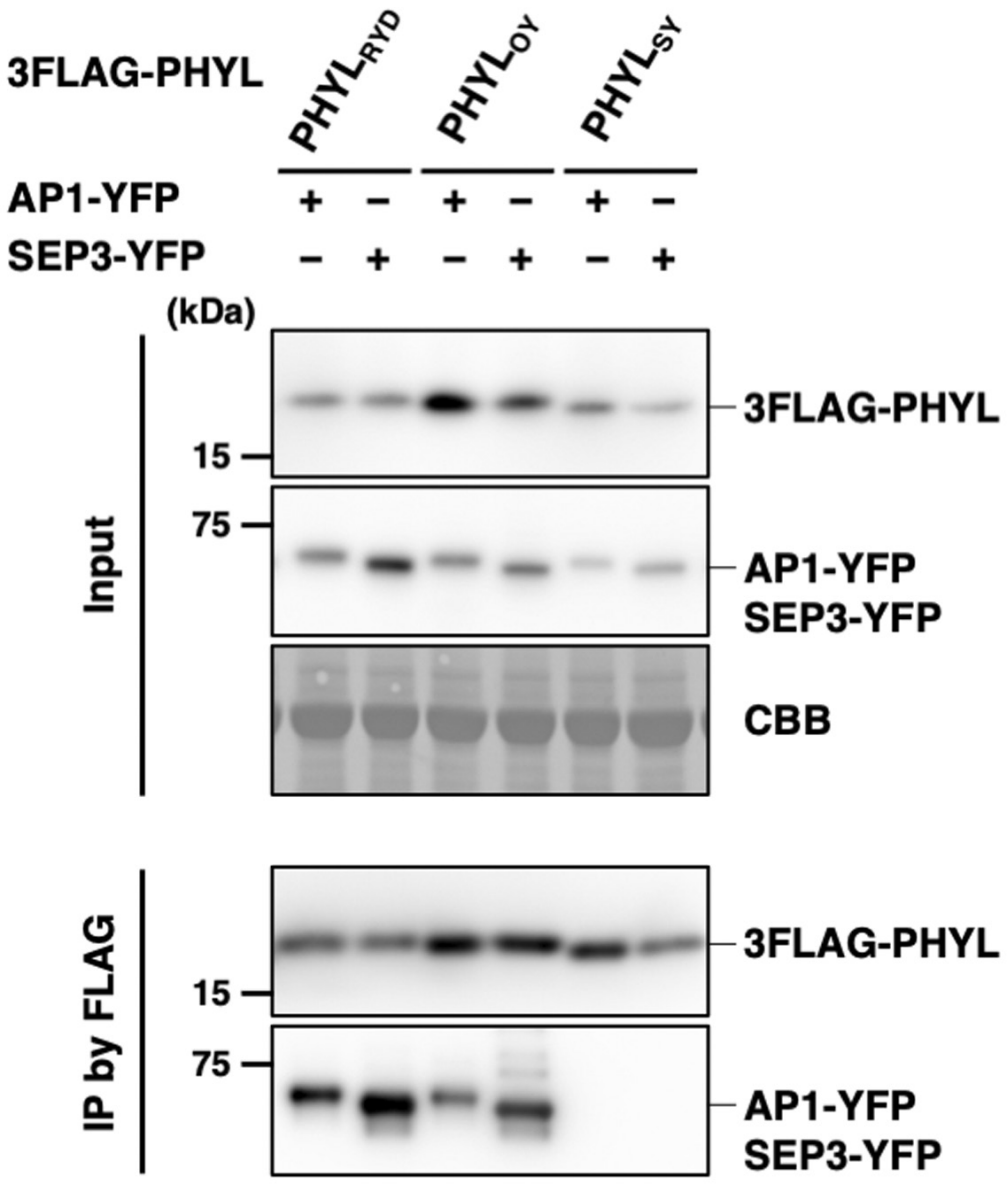
PHYL_RYD_ interacts with AP1 and SEP3 in planta. MTF‐binding affinity of phyllogen homologues was examined by co‐immunoprecipitation assay in planta. 3FLAG‐PHYL and MTF‐YFP were co‐expressed in *Nicotiana benthamiana* leaves by agroinfiltration. Total proteins were extracted 36 h post‐infiltration (Input), and 3FLAG‐PHYL was immunoprecipitated using an anti‐FLAG antibody (IP by FLAG). Accumulation of 3FLAG‐PHYL and MTF‐YFP was evaluated by immunoblotting using anti‐FLAG and anti‐GFP antibodies, respectively. Coomassie brilliant blue (CBB) staining is shown as a loading control.

To investigate the molecular mechanism underlying the narrow range of MTFs degraded by PHYL_RYD_, the MTF‐binding affinity of PHYL_RYD_ was examined in yeast two‐hybrid (Y2H) assays (Table 1 and Figure S4). GAL4 activation domain (AD)‐fused MTF and DNA‐binding domain (BD)‐fused phyllogen were co‐expressed in yeast cells, and their interaction was evaluated in terms of yeast growth on selective media. The results indicated that PHYL_RYD_ bound to all the tested *Arabidopsis* MTFs to the same extent, regardless of the degradation activity. Similarly, although PHYL_RYD_ showed different degrees of binding affinity depending on the type of *Oryza* MTFs, these differences did not correlate with its MTF degradation activity (Table 1). This result was in contrast to previous reports that phyllogens lacking MTF degradation activity show impaired MTF‐binding affinity (Iwabuchi et al., 2019, 2020; Liao et al., 2019). Furthermore, the interaction between MTF and PHYL_RYD_ was evaluated in planta by co‐immunoprecipitation assays (Figure 4). AP1 and SEP3 were used as representatives of PHYL_RYD_‐degradable MTFs and non‐PHYL_RYD_‐degradable MTFs, respectively. We co‐expressed 3FLAG‐fused phyllogens (3FLAG‐PHYL_RYD_, PHYL_OY_ or PHYL_SY_) and yellow fluorescent protein (YFP)‐fused MTFs (AP1‐YFP or SEP3‐YFP) in *N. benthamiana* leaves, and 3FLAG‐PHYL was immunoprecipitated by an anti‐FLAG antibody. AP1‐YFP and SEP3‐YFP were co‐immunoprecipitated with 3FLAG‐PHYL_OY_, but not with 3FLAG‐PHYL_SY_. This result implies correspondence between MTF‐binding affinity and MTF degradation activity in these phyllogens, as reported previously (Iwabuchi et al., 2020). In contrast, both AP1‐YFP and SEP3‐YFP were co‐immunoprecipitated as efficiently with 3FLAG‐PHYL_RYD_ as with 3FLAG‐PHYL_OY_, indicating that PHYL_RYD_ retains affinity to non‐PHYL_RYD_‐degradable MTFs in planta. These results clarified that the low MTF degradation activity of PHYL_RYD_ was not attributable to a defect in MTF‐binding affinity.

**TABLE 1:** PHYL_RYD_ interacts with non‐degradable MADS box transcription factors (MTFs) in yeast.

DNA‐binding domain (BD)	Activation domain (AD)										
	Empty	*Arabidopsis* MTF					*Oryza* MTF				
		AP1	SEP1	SEP2	SEP3	SEP4	OsMADS14	OsMADS15	OsMADS18	OsMADS5	OsMADS8
Empty	−^a^	−^b^	−^c^	−^c^	−^b^	−^c^	−^d^	−	−	+	−^d^
PHYL_RYD_	−	++	++	++	++	++	++	+	+	+	++
PHYL_OY_	−^c^	++^a^	+++^c^	+++^c^	+++^c^	+++^c^	++^d^	+	+	+	++^d^

*Note*: The symbols indicate the growth of yeast co‐expressing activation domain (AD)‐fused MTF and DNA‐binding domain (BD)‐fused phyllogen on the selective media (−LWAH, −LWH + 3AT, −LWH and −LW): +++ the yeast grew on all the four media; ++ the yeast grew on −LWH + 3AT, −LWH and −LW; + the yeast grew on −LWH and −LW; − the yeast grew only on −LW. A higher number of + indicates stronger interaction between MTF and phyllogen in yeast, while − indicates that no significant interaction was detected. Raw data of yeast growth are shown in Figure S4. Several results were previously reported in ^a^Iwabuchi et al. (2020), ^b^Maejima, Iwai, et al. (2014), ^c^Iwabuchi et al. (2019), and ^d^Kitazawa et al. (2017).

### 2.5 RAD23 recruitment ability of PHYL_RYD_

**FIGURE 5:**
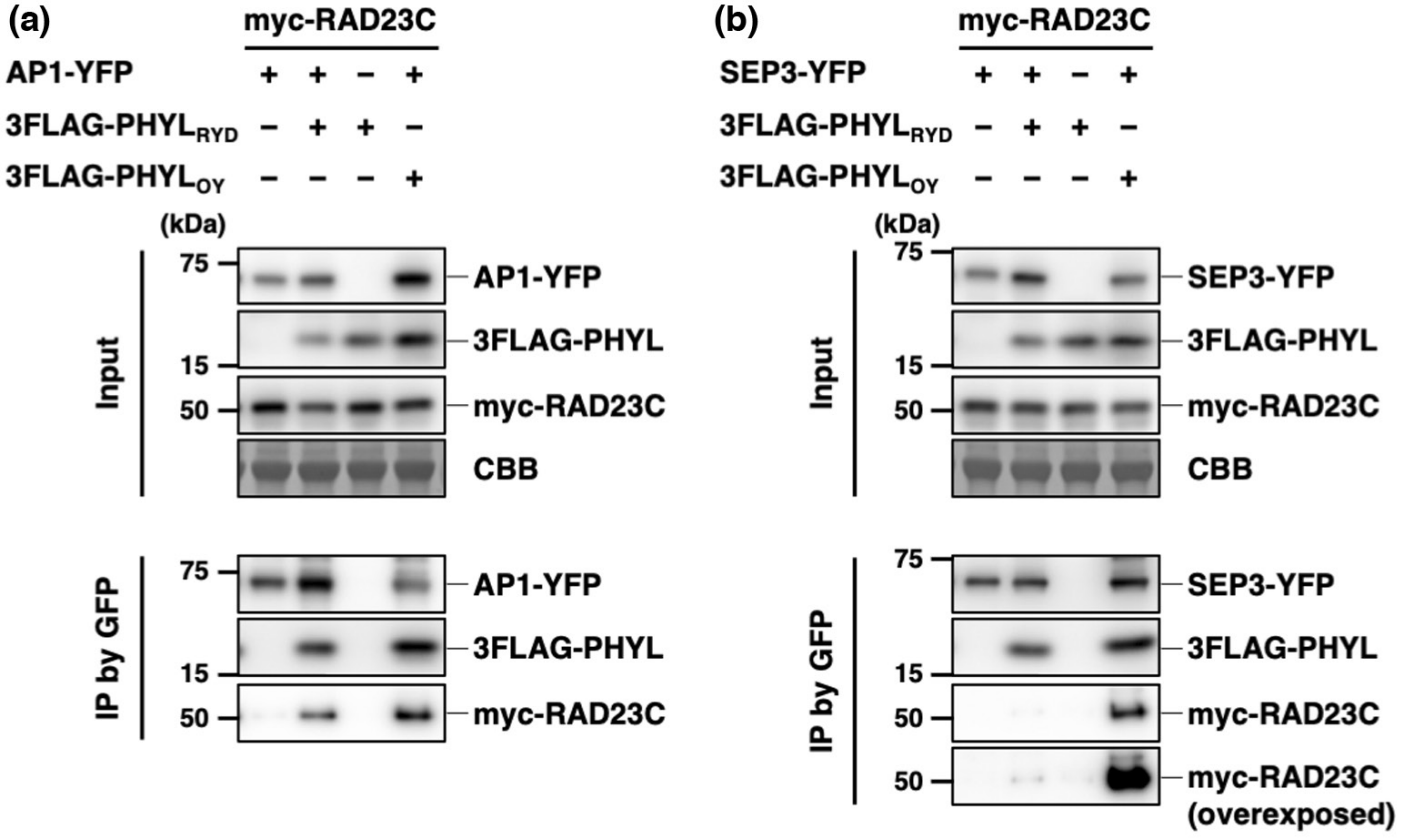
AP1/PHYL_RYD_ complex efficiently recruits RAD23C, but SEP3/PHYL_RYD_ complex does not. (a) Co‐immunoprecipitation assays to examine ternary interaction between AP1, PHYL_RYD_ and RAD23C. AP1‐YFP, 3FLAG‐PHYL_RYD_ and myc‐RAD23C were co‐expressed in *Nicotiana benthamiana* leaves by agroinfiltration. Total proteins were extracted 36 h post‐infiltration (Input), and AP1‐YFP was immunoprecipitated using an anti‐GFP antibody (IP by GFP). Accumulation of AP1‐YFP, 3FLAG‐PHYL and myc‐RAD23C was analysed by immunoblotting using anti‐GFP, anti‐FLAG and anti‐myc antibodies, respectively. Coomassie brilliant blue (CBB) staining is shown as a loading control. (b) Co‐immunoprecipitation assays using SEP3‐YFP, 3FLAG‐PHYL and myc‐RAD23C. The experimental conditions were as described in (a).

In addition to floral MTFs, phyllogen also binds to RAD23, a proteasomal shuttle protein (Kitazawa et al., 2022; MacLean et al., 2014). Y2H assays confirmed that PHYL_RYD_ could interact with RAD23 of both *A. thaliana* and *O. sativa* in yeast cells (Table S1 and Figure S4). Kitazawa et al. (2022) proposed that the formation of the MTF/phyllogen complex triggers RAD23 recruitment in planta, resulting in the proteasomal degradation of the target MTF. Based on this model, we hypothesized that the low MTF degradation activity of PHYL_RYD_ was caused by impaired RAD23 recruitment ability of the MTF/PHYL_RYD_ complex. To compare the RAD23 recruitment ability between AP1 (PHYL_RYD_‐degradable MTF) and SEP3 (non‐PHYL_RYD_‐degradable MTF) when forming a complex with PHYL_RYD_, co‐immunoprecipitation assays were performed in planta (Figure 5). AP1‐YFP and myc‐fused RAD23C (myc‐RAD23C) were transiently expressed with and without 3FLAG‐PHYL_RYD_ in *N. benthamiana* leaves, and AP1‐YFP was immunoprecipitated using an anti‐green fluorescent protein (GFP) antibody. In the presence of 3FLAG‐PHYL_RYD_, both 3FLAG‐PHYL_RYD_ and myc‐RAD23C were co‐immunoprecipitated with AP1‐YFP (Figure 5a). This result indicates that the AP1/PHYL_RYD_ complex can recruit RAD23C. In contrast, when SEP3‐YFP was used instead of AP1‐YFP, 3FLAG‐PHYL_RYD_ was efficiently co‐immunoprecipitated with SEP3‐YFP, whereas myc‐RAD23C was not (Figure 5b). This result demonstrates that the SEP3/PHYL_RYD_ complex cannot recruit RAD23C, which is consistent with the inability of PHYL_RYD_ to degrade SEP3 (Figure 3a). Notably, when PHYL_OY_ was used instead of PHYL_RYD_, both the AP1/PHYL_OY_ complex and the SEP3/PHYL_OY_ complex were able to recruit RAD23C (Figure 5). Together, these findings show that RAD23 recruitment ability of the MTF/phyllogen complex differs depending on the combination of MTF and phyllogen and corresponds to the degradation activity.

## 3 DISCUSSION

### 3.1 Novel mechanism for the target specificity of phyllogens in MTF degradation

**FIGURE 6:**
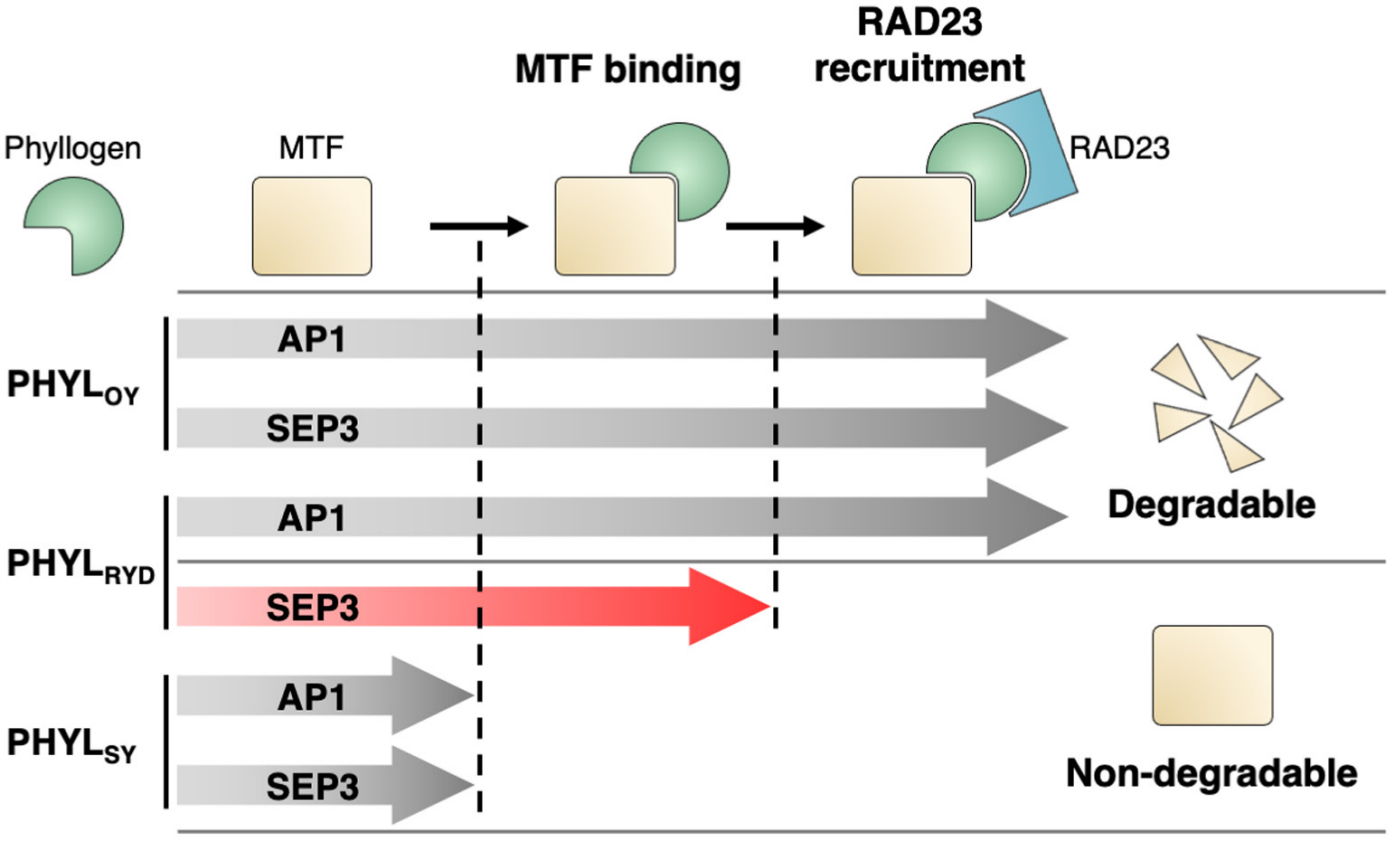
MADS box transcription factor (MTF)‐binding affinity and RAD23 recruitment ability cooperatively regulate target specificity of phyllogens. Phyllody‐inducing phyllogens including PHYL_OY_ first bind to target MTFs such as AP1 and SEP3, and then recruit RAD23 to induce MTF degradation. In contrast, non‐phyllody‐inducing phyllogens including PHYL_SY_ have an impaired MTF‐binding affinity and cannot efficiently degrade floral MTFs. PHYL_RYD_ degrades a narrower range of floral MTFs than PHYL_OY_ because RAD23 recruitment occurs only when PHYL_RYD_ has recognized its target MTFs such as AP1.

The existing body of research on various effector proteins suggests that effector target specificity is often determined by its binding properties in relation to host proteins (Huang et al., 2021; Oikawa et al., 2020; Pecher et al., 2019; Tanaka et al., 2019). For phyllogen as well, several findings have indicated that the MTF‐binding affinity of phyllogen determines its target specificity for MTF degradation (Iwabuchi et al., 2019, 2020; Liao et al., 2019; MacLean et al., 2014). Phyllogen homologues that degrade floral MTFs (e.g., PHYL_OY_ in Figure 6) first bind to a target MTF, and the MTF/phyllogen complex then recruit RAD23 to induce MTF degradation (Kitazawa et al., 2022). On the contrary, phyllogen homologues that cannot degrade floral MTFs (e.g., PHYL_SY_ in Figure 6) do not bind to those MTFs (Iwabuchi et al., 2020).

The present study analysed PHYL_RYD_, a phyllogen homologue of RYD phytoplasma, and revealed a novel mechanism for the degradation target specificity of phyllogen in which the MTF degradation specificity is determined by RAD23 recruitment ability rather than MTF‐binding affinity. PHYL_RYD_ retains residues that are important for MTF binding (Figure 1) and binds to non‐target MTFs that PHYL_RYD_ is unable to degrade (Table 1 and Figure 4). Interestingly, although the complex of PHYL_RYD_ and AP1 (PHYL_RYD_‐degradable MTF) efficiently recruited RAD23, the complex of PHYL_RYD_ and SEP3 (non‐PHYL_RYD_‐degradable MTF) could not (Figure 5). These results elucidated that the RAD23 recruitment ability of the MTF/PHYL_RYD_ complex differs according to the type of interacting MTF, resulting in a difference in degradation activity (Figure 6). Given that phyllogen homologues share the same mechanism for MTF degradation (Kitazawa et al., 2022), the finding that RAD23 recruitment ability determines the MTF degradation activity would be applicable to other phyllogens than PHYL_RYD_. Thus, the target specificity of phyllogen in MTF degradation is probably regulated by two steps: the MTF‐binding affinity of phyllogen and the RAD23 recruitment ability of the resulting MTF/phyllogen complex (Figure 6). The discovery of this two‐step target regulation mechanism also implies that caution is warranted when discussing the target specificity and functions of effector proteins solely on the basis of their binding affinity to host proteins.

### 3.2 Elucidating the molecular mechanism of RAD23 recruitment

Kitazawa et al. (2022) showed that the MTF/phyllogen complex directly binds to RAD23 without ubiquitin, leading to the proteasomal degradation of host MTF in a ubiquitin‐independent manner. This finding is noteworthy, as previous research has indicated that effector‐mediated proteasomal degradation of host factors typically requires their ubiquitination (Ashida et al., 2014; Lin & Machner, 2017). To further elucidate this unique target degradation mechanism, the next step is to investigate how the MTF/phyllogen complex interacts with RAD23. Phyllogens recognize the K domain of MTF (MTF_K_) and a ubiquitin‐associated 2 (UBA2) domain of RAD23, a domain that originally binds to ubiquitin chains (Kitazawa et al., 2022). Because neither phyllogen nor MTF_K_ shows structural similarity with ubiquitin, and no residues have been identified that are important for the interaction with UBA2, it is difficult to predict how the UBA2 domain binds to the MTF_K_/phyllogen complex. The results of this study revealed that SEP3/PHYL_RYD_ has a much lower affinity to RAD23C compared with the AP1/PHYL_RYD_ and SEP3/PHYL_OY_ complexes (Figure 5), which suggests that comparing the sequences and structures of AP1_K_ and SEP3_K_, or those of PHYL_RYD_ and PHYL_OY_, would help to identify the key residues or structures responsible for the interaction with RAD23 and shed light on how the MTF/phyllogen complex recruits RAD23.

### 3.3 Importance of evaluating MTF degradation to analyse phyllogen functions

Several studies have raised the possibility that phyllogen may possess additional functions beyond its capacity to induce phyllody. Phyllogen expression has been shown to enhance the colonization of insect vectors, although the underlying molecular mechanism remains to be determined (Orlovskis & Hogenhout, 2016). Additionally, comprehensive Y2H assays have demonstrated that phyllogen can interact not only with floral MTFs but also with several other non‐floral MTFs involved in diverse plant developmental processes (Correa Marrero et al., 2023; MacLean et al., 2014). Moreover, the binding affinity to the non‐floral MTFs can differ among phyllogen homologues (Kitazawa et al., 2023). However, whether phyllogen can degrade these non‐floral MTFs remains to be clarified. This study elucidated that MTFs interacting with phyllogen are not necessarily degraded, emphasizing the importance of examining MTF degradation activity rather than MTF‐binding affinity. Comprehensive MTF degradation assays will significantly advance our understanding of the functions of phyllogen.

### 3.4 Functional characteristics of PHYL_RYD_

Previously characterized phyllogens belonging to the phyl‐A, C and D groups (e.g., PHYL_OY_) efficiently degrade AP1 and SEP1–4 and cause phyllody in *A. thaliana*, whereas phyllogens in the phyl‐B group (e.g., PHYL_SY_) do not degrade either AP1 or SEP1–4 and do not induce visible flower abnormalities (Iwabuchi et al., 2020). When compared to these phyllogens, PHYL_RYD_ is unique in both the narrow range of target MTFs (Figure 3) and the induction of the intermediate phyllody phenotypes (Figures 2 and S2).

The intermediate phyllody observed in PHYL_RYD_‐expressing *A. thaliana* probably reflects its narrower degradation targets. *AP1* gene encodes the only A‐class MTF in *A. thaliana*, and *ap1* single mutants show various abnormalities in sepals and petals but seldom induce stamen and pistil disorders (Chuang & Meyerowitz, 2000; Gregis et al., 2006). Similarly, PHYL_RYD_‐expressing plants mainly exhibited abnormalities in sepals and petals, whereas stamens and pistils were less affected (Figure 2). This finding suggests that AP1 degradation could be involved in the induction of floral abnormalities by PHYL_RYD_. On the contrary, *SEP1–4* genes encoding E‐class MTFs in *A. thaliana* are functionally redundant, and the conversion of floral organs into sepal‐ or leaf‐like organs is observed only in triple and quadruple *sep* mutants (Ditta et al., 2004; Irish, 2017). Therefore, the compromised phyllody‐inducing activity of PHYL_RYD_ can be attributed to its low degradation ability for SEP3 and SEP4. Besides, floral MTFs are widely conserved in angiosperms (Irish, 2017), and phyllogens can degrade floral MTFs in various plant species (Kitazawa et al., 2017). Although the degradation activity of PHYL_RYD_ for the floral MTFs of *N. benthamiana* was not examined in this study, the compromised phyllody‐inducing activity of PHYL_RYD_ observed in *N. benthamiana* (Figures 2 and S2) may reflect its narrow range of target MTFs, as observed in *A. thaliana*.

### 3.5 Considerations on the function of PHYL_RYD_ in rice

Certain phytoplasma strains lack a functional phyllogen due to the absence or pseudogenization of the phyllogen gene in the genome (Aurin et al., 2020; Iwabuchi et al., 2020; Maejima, Iwai, et al., 2014). In contrast, PHYL_RYD_ retains the full open reading frame, despite accumulating multiple mutations (Figure 1a). Therefore, there is an interest in the biological functions of PHYL_RYD_. The flowers and inflorescences of grass species including rice are completely different from those of other plant species such as *A. thaliana* and *N. benthamiana*: one or several flowers lacking sepals and petals compose one small spikelet, and an aggregate of a substantial number of spikelets composes one ear (Yamaguchi & Hirano, 2006). Based on this characteristic, we propose two hypotheses regarding the biological implication of the narrower range of target MTFs of PHYL_RYD_. One hypothesis is that PHYL_RYD_ is gradually losing its function and may not significantly contribute to RYD phytoplasma infection. Su et al. (2011) reported that phytoplasmas infecting periwinkle plants accumulate to a greater extent in flowers with phyllody symptoms than in surrounding leaves; they proposed that phyllody might help to increase the phytoplasma population. However, a grass spikelet has no sepals or petals and is too tiny to significantly increase the phytoplasma population by inducing phyllody. In addition, no full‐length conserved phyllogen has been found in phytoplasma strains infecting cereal grasses, except for PHYL_RYD_ (Iwabuchi et al., 2020; Zhu et al., 2017). These considerations suggest that gene loss or functional impairment of phyllogens may have occurred in grass‐infecting phytoplasmas, possibly because phyllogens were dispensable due to the unique inflorescence architecture of their host plants.

An alternative hypothesis is that PHYL_RYD_ selectively degrades certain *Oryza* MTFs that regulate inflorescence development. Among AP1‐like *Oryza* MTFs, PHYL_RYD_ degrades OsMADS14 and OsMADS15, but not OsMADS18 (Figure 3b). Despite the high degree of sequence similarity among these three *Oryza* MTFs, their functions are distinct. *OsMADS18* is expressed in most tissues and is involved in diverse developmental processes, including abscisic acid response, seed germination and flowering time (Wu et al., 2017). In contrast, *OsMADS14* and *OsMADS15* are mainly expressed in the inflorescence to specify the inflorescence meristem identity, and loss of their functions leads to spikelet sterility or failure to produce ears (Wu et al., 2017). As rice plants infected by RYD phytoplasma exhibit spikelet sterility and reduced ear numbers (Komori, 1965), PHYL_RYD_ may contribute to these symptoms by degrading OsMADS14 and OsMADS15. Furthermore, rice mutants with spikelet sterility exhibit increased tillers, sustained high photosynthesis activity and delayed senescence (Kato et al., 2004, 2006). These secondary changes may enhance the fitness of RYD phytoplasma by improving the trophic conditions in sieve tissue and increasing the chance of transmission by its insect vector. Further investigation into the biological functions of PHYL_RYD_ in rice will advance our understanding of the functional diversification of the phyllogen family beyond the induction of phyllody symptoms.

## 4 EXPERIMENTAL PROCEDURES

### 4.1 Materials

*A. thaliana* ecotype Col‐0 was maintained in a growth chamber under 16‐h light/8‐h dark conditions at 22°C. *N. benthamiana* was grown under natural light conditions at 25°C before use and maintained in a growth chamber with 16‐h light/8‐h dark conditions at 25°C during the experiments.

The genes, plasmids and primers used in this study are listed in Tables S2–, S4, respectively. Genes used in the experiments were cloned into pENTA (Himeno et al., 2010), a pUC19‐based plasmid containing the attL1‐MCS‐attL2 sequence of pENTR1A. As summarized in Table S3, some genes were cloned in previous reports (Iwabuchi et al., 2019, 2020; Kitazawa et al., 2017, 2022, 2023; Maejima, Iwai, et al., 2014), whereas others were newly cloned into pENTA. Briefly, *OsMADS5*, *OsMADS18* and *OsRAD23c* were amplified from the RNA of *O. sativa* ‘Koshihikari’ by RT‐PCR using appropriate primers (Table S4). A DNA fragment of *OsMADS15* was synthesized by Eurofins Genomics and PCR‐amplified using OsMADS15‐to‐pENTA‐F and R primers. The amplified fragment and pENTA digested with SalI and EcoRV (New England Biolabs) were assembled using the seamless ligation cloning extract (SLiCE) method (Motohashi, 2015). The genes in pENTA were subcloned into appropriate vectors, as described below.

### 4.2 Sequence‐based analyses of PHYL_RYD_

Sequence identity between PHYL_RYD_ and other phyl‐A group phyllogens including the putative signal peptide was calculated using the Sequence Demarcation Tool v. 1.2 (Muhire et al., 2014) with the MUSCLE algorithm (Edgar, 2004).

The three‐dimensional protein structure of the putative secreted region of PHYL_RYD_ was predicted by ColabFold (Mirdita et al., 2022). The parameters were as follows: use_amber = false, template_mode = none, msa_mode = MMseqs2 (UniRef+Environmental), pair_mode = unpaired+paired, model_type = AlphaFold2‐ptm and num_recycles = 3. The predicted structure of PHYL_RYD_ was visualized with UCSF ChimeraX v. 1.4 (https://www.rbvi.ucsf.edu/chimerax/).

### 4.3 Observations of floral phenotypes induced by phyllogen

A modified TRV‐based gene expression vector (Iwabuchi et al., 2019) was used for systemic gene expression. PHYL_RYD_ and pTRV2 were amplified with primer pairs 2A‐RYD‐F/pTRV2‐RYD‐R and pTRV2‐1173‐1193F/2A‐R, respectively (Table S4). Both amplified fragments were assembled using the GeneArt Seamless Cloning and Assembly Kit (Invitrogen). Plasmid transformation into *Agrobacterium tumefaciens* EHA105 (Hood et al., 1993) and preparation of *Agrobacterium* suspensions for agroinfiltration were performed as described previously (Takahashi et al., 2006). *A. tumefaciens* lines transformed with p19, pTRV1, pTRV2‐PHYL_OY_ and pTRV2‐empty were produced in previous studies (Iwabuchi et al., 2019, 2020). *Agrobacterium* suspensions of p19, pTRV1 and pTRV2, each at an optical density at 600 nm (OD_600_) of 0.1, were mixed at a ratio of 1:1:5 and co‐infiltrated into 3‐week‐old *A. thaliana* and 4‐week‐old *N. benthamiana* leaves. At approximately 3 weeks (*A. thaliana*) or 5 weeks (*N. benthamiana*) after TRV vector infiltration, floral phenotypes were observed. The criteria for phenotype scoring of *N. benthamiana* flowers are described in Figure S2. TRV infection in flowers was confirmed by amplifying the phyllogen‐insertion region using RT‐PCR with trv‐F and trv‐R primers, as described previously (Iwabuchi et al., 2019). Four independent experiments were conducted for both *A. thaliana* and *N. benthamiana*.

### 4.4 MTF degradation assay

To examine the MTF degradation activity of phyllogen in *N. benthamiana*, protein expression vectors of N‐terminal myc‐fused MTF (myc‐MTF) and N‐terminal 3FLAG‐fused phyllogen (3FLAG‐PHYL) were constructed. Using Gateway LR Clonase II enzyme mix (Invitrogen), genes cloned in pENTA were subcloned into a pEarleyGate vector series: three *OsMADS* genes cloned in this study, *OsMADS8* and *OsMADS14* (Kitazawa et al., 2017) were subcloned into pEarleyGate203 (Earley et al., 2006) for myc‐OsMADS expression, and *PHYL*_*RYD*_ (Kitazawa et al., 2023) was subcloned into pEarleyGateN3 × FLAG (Iwabuchi et al., 2019) for 3FLAG‐PHYL_RYD_ expression. Vectors expressing myc‐fused MTFs of *A. thaliana*, 3FLAG‐PHYL_OY_ and 3FLAG‐PHYL_SY_ were constructed previously (Iwabuchi et al., 2019, 2020). Plasmid transformation and preparation of *Agrobacterium* suspensions were conducted as described above. *Agrobacterium* suspensions transformed with p19, myc‐MTF and 3FLAG‐PHYL (OD_600_ = 1.0 for each) were mixed at a ratio of 1:10:1. For the mixture without 3FLAG‐PHYL, an *Agrobacterium*‐free infiltration buffer (Takahashi et al., 2006) was added instead of the 3FLAG‐PHYL suspension. Mixtures with and without 3FLAG‐PHYL were infiltrated on the right and left side, respectively, of the same leaf of a 4‐week‐old *N. benthamiana* plant. At 36 h after infiltration, leaf disks of the infiltrated area were collected and frozen immediately in liquid nitrogen. Protein extraction and western blot analyses were carried out as described previously (Kitazawa et al., 2017). Proteins were detected by anti‐myc (4A6; Millipore), anti‐FLAG (F1804; Sigma‐Aldrich) and anti‐GFP (7.1 and 13.1; Roche) antibodies. For each combination of MTF and phyllogen, at least two replicates were prepared in each experiment and at least two independent experiments were conducted.

### 4.5 In planta co‐immunoprecipitation assay

For C‐terminal YFP‐fused AP1 (AP1‐YFP) expression, AP1 in pENTA (Maejima, Iwai, et al., 2014) was subcloned into pEarleyGate101 (Earley et al., 2006), and the plasmid was transformed into *A. tumefaciens*, as described above. *A. tumefaciens* lines expressing SEP3‐YFP or myc‐RAD23C were described previously (Kitazawa et al., 2022; Maejima, Iwai, et al., 2014). *Agrobacterium* suspensions (OD_600_ = 1.0 for each) were mixed at the following ratios: p19:3FLAG‐PHYL:MTF‐YFP = 1:5:5 for anti‐FLAG immunoprecipitation and p19:MTF‐YFP:3FLAG‐PHYL:myc‐RAD23C = 3:10:10:10 for anti‐GFP immunoprecipitation. The mixtures were infiltrated into entire leaves of 4‐week‐old *N. benthamiana*, and the leaves were collected 36 h after infiltration. Protein extraction, sample incubation with antibody beads and bead washing were conducted as described previously (Iwabuchi et al., 2020) using EZview Red ANTI‐FLAG M2 Affinity Gel (Sigma‐Aldrich) and GFP‐Trap Agarose (ChromoTek) for anti‐FLAG and anti‐GFP immunoprecipitation, respectively. In anti‐FLAG immunoprecipitation, proteins immunoprecipitated with beads were eluted with 30 μL of a 1 × RIPA buffer containing 200 ng/μL 3 × FLAG peptide (Sigma‐Aldrich) at 4°C for 30 min with gentle tapping every 10 min. After centrifugation, the bead‐free supernatant was retrieved, mixed with 10 μL of 4 × sodium dodecyl sulphate (SDS) sample buffer (Kitazawa et al., 2022) and incubated for 5 min at 95°C (IP by FLAG). For anti‐GFP immunoprecipitation, the washed beads were suspended in 80 μL of 2 × SDS sample buffer and heat‐denatured for 5 min at 95°C. The mixture was centrifuged, and the bead‐free supernatant was retrieved (IP by GFP). Western blot analyses were conducted as described above. Two and four independent experiments were performed for anti‐FLAG and anti‐GFP immunoprecipitation, respectively.

### 4.6 Y2H assay

The Matchmaker GAL4 Two‐Hybrid System 3 kit (Clontech) was used to evaluate protein–protein interactions in yeast cells. *OsMADS5*, *OsMADS8* and *OsMADS18* genes in pENTA were PCR‐amplified using their respective forward primers and pENTA‐to‐pGADT7‐EcoRI‐R reverse primers (Table S4). *OsRAD23c* was amplified with OsRAD23c‐to‐pGADT7‐F and R primers. Each amplified fragment and pGADT7 vector (Clontech) digested with NdeI and EcoRI‐HF (New England Biolabs) were ligated using the SLiCE method to construct AD‐fused OsMADSs and OsRAD23c. Other genes were previously cloned into pGADT7 and pGBKT7 vectors, as shown in Table S3 (Iwabuchi et al., 2019, 2020; Kitazawa et al., 2017, 2022, 2023; Maejima, Iwai, et al., 2014). Appropriate pairs of pGADT7 and pGBKT7 vectors were co‐transformed into yeast strain AH109, and protein interaction was evaluated on selective media, as described previously (Kitazawa et al., 2017).

## CONFLICT OF INTEREST STATEMENT

The authors declare that they have no competing interests.

## Supporting information


**Figure S1.** There is an evolutionary distance between PHYL_RYD_ and other phyl‐A group phyllogens. Amino acid sequences of phyllogens including the putative signal peptide were aligned by MUSCLE algorithm (Edgar, 2004) using MEGA v. 10.1.8 (Kumar et al., 2018). Using all sites, the phylogenetic tree was constructed by the maximum likelihood method based on the Jones–Taylor–Thornton model with gamma distribution. The number at each branch indicates the bootstrap value (%) obtained from 1000 replicates (only values >70% are shown). PHYL_RYD_ is shown in bold. Phyllogens which were confirmed to induce phyllody (Iwabuchi et al., 2020; MacLean et al., 2014; Tokuda et al., 2023) are indicated by red arrowheads. Three phyllogens in the phyl‐B group (PHYL_SY_, PHYL_MD_, and PHYL_PvWB_) were used as outgroups but were omitted in the tree for simplicity. Accession numbers of the genes are shown in Table S2.

**Figure S2.** PHYL_RYD_ rarely induces floral abnormality in *Nicotiana benthamiana*. (a) Representative image of each floral phenotype observed in phyllogen‐expressing *N. benthamiana* plants. Floral phenotypes were classified into four levels based on the following criteria: (normal) The flower shows no symptom; (malformation) The shape or number of floral organ(s) is affected but floral organ identity is maintained; (phyllody) The identity of floral organ(s) is altered but the floral determinacy is not lost; (phyllody & proliferation) The organ identity and the structure of the entire flower are disordered, and a new shoot was produced due to loss of floral determinacy. Sepals and petals are indicated as se and pe, respectively. Bars = 1 cm. (b) The frequency of each floral phenotype. Approximately 5 weeks after TRV vector inoculation, all mature flowers produced on the main stem were observed. The numbers in parentheses indicate the total number of plants observed in four independent experiments.

**Figure S3.** PHYL_RYD_ does not target specific MTF clades for degradation. Amino acid sequences of *Arabidopsis* and *Oryza* MTFs were aligned by MUSCLE algorithm (Edgar, 2004) using MEGA v. 10.1.8 (Kumar et al., 2018). Gaps in the alignment were eliminated by complete deletion. The phylogenetic tree was constructed based on neighbour‐joining method with gamma distribution. The number at each branch indicates the bootstrap value (%) obtained from 1000 replicates (only values >70% are shown). Whether the MTF is degraded or not by PHYL_RYD_ and PHYL_OY_ is indicated on the right side of the phylogenetic tree. AGAMOUS (AG) belonging to *Arabidopsis* C‐class MTF was used as an outgroup. Accession numbers of the genes are shown in Table S2. *Reported in Maejima, Iwai, et al. (2014).

**Figure S4.** Growth of yeast on selective media in yeast two‐hybrid assays. MTF and RAD23 were cloned into the pGADT7 vector to fuse AD while phyllogen was cloned into the pGBKT7 vector to fuse BD. Because the pGADT7 and pGBKT7 vectors contain leucine (L) and tryptophan (W) synthesis enzymes, respectively, yeast transformed with both plasmids can grow on a selective medium without these nutrients (−LW). Furthermore, depending on the degree of interaction between AD‐fused and BD‐fused proteins, gene expression of histidine (H) and adenine (A) synthesis enzymes is induced, allowing yeast to grow on media with more stringent selection conditions (−LW < −LWH < −LWH + 3AT < −LWAH). Several results were previously reported in ^a^Iwabuchi et al. (2020), ^b^Maejima, Iwai, et al. (2014), ^c^Iwabuchi et al. (2019), ^d^Kitazawa et al. (2017), and ^e^Kitazawa et al. (2022).

**Table S1.** PHYL_RYD_ interacts with RAD23 in yeast. The symbols indicate the growth of yeast co‐expressing AD‐fused RAD23 and BD‐fused phyllogen on the selective media (−LWAH, −LWH + 3AT, −LWH, and − LW): +++ the yeast grew on all the four media; ++ the yeast grew on −LWH + 3AT, −LWH, and − LW; + the yeast grew on −LWH and − LW; − the yeast grew only on −LW. A higher number of + indicates stronger interaction between RAD23 and phyllogen in yeast, while − indicates that no significant interaction was detected. Raw data of yeast growth are shown in Figure S4. Several results were previously reported in ^a^Iwabuchi et al. (2020), ^b^Iwabuchi et al. (2019), and ^c^Kitazawa et al. (2022).

**Table S2.** Genes used in the study.

**Table S3.** Plasmids used in the study.

**Table S4.** Primers used in the study.

## Data Availability

The data that support the findings of this study are available from the corresponding author upon reasonable request.
