# Supplementary figures and images for: Target degradation specificity of phytoplasma effector phyllogen is regulated by the recruitment of host proteasome shuttle protein
Source: Mol Plant Pathol. 2023 Dec 17;25(1):e13410. doi: 10.1111/mpp.13410 (PMC10799209; doi:10.1111/mpp.13410)

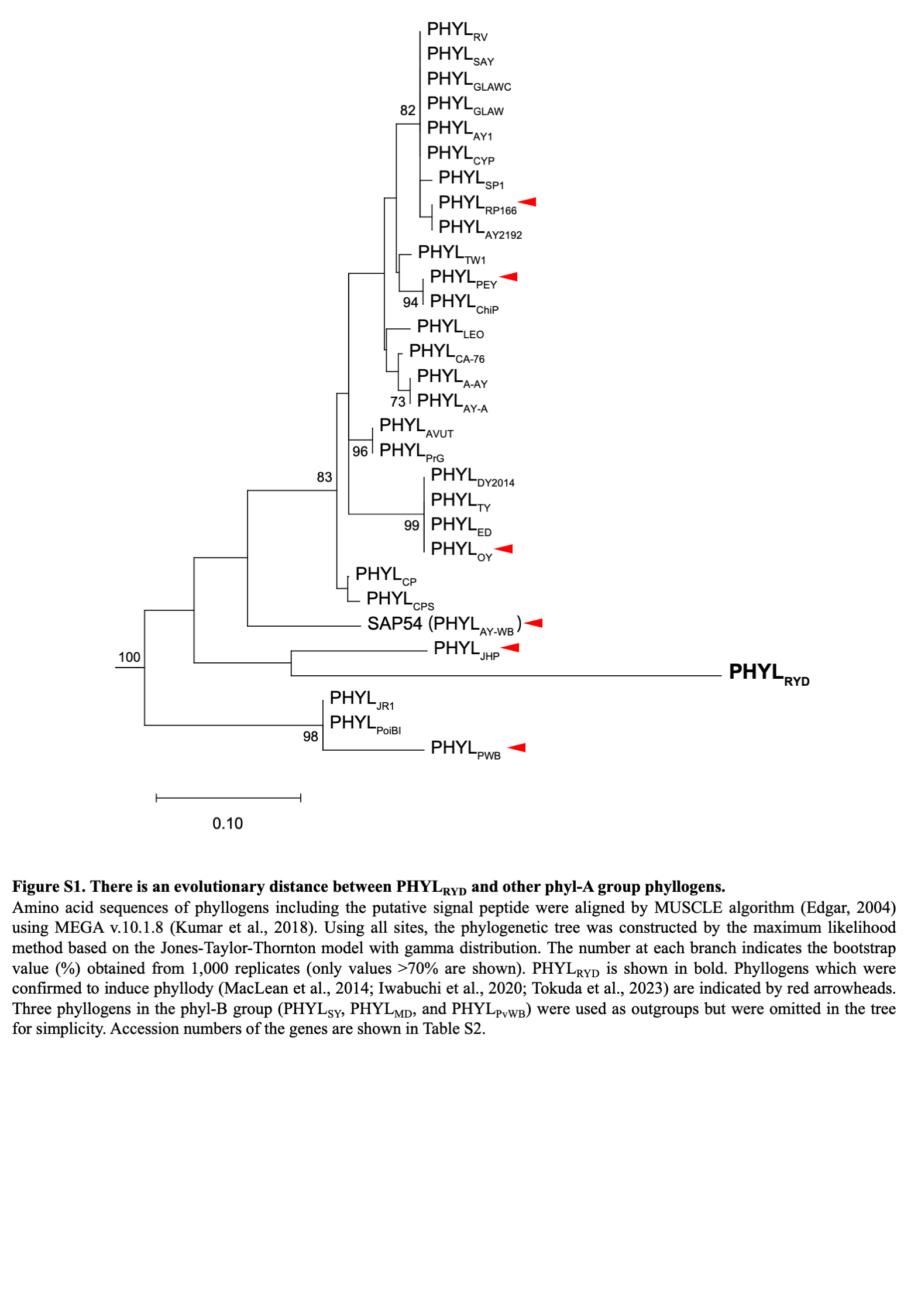

Supplement: Supplementary file 1 — Figure S1. There is an evolutionary distance between PHYLRYD and other phyl‐A group phyllogens. Amino acid sequences of phyllogens including the putative signal peptide were aligned by MUSCLE algorithm (Edgar, 2004) using MEGA v. 10.1.8 (Kumar et al., 2018). Using all sites, the phylogenetic tree was constructed by the maximum likelihood method based on the Jones–Taylor–Thornton model with gamma distribution. The number at each branch indicates the bootstrap value (%) obtained from 1000 replicates (only values >70% are shown). PHYLRYD is shown in bold. Phyllogens which were confirmed to induce phyllody (Iwabuchi et al., 2020; MacLean et al., 2014; Tokuda et al., 2023) are indicated by red arrowheads. Three phyllogens in the phyl‐B group (PHYLSY, PHYLMD, and PHYLPvWB) were used as outgroups but were omitted in the tree for simplicity. Accession numbers of the genes are shown in Table S2. [file MPP-25-e13410-s002.tiff]

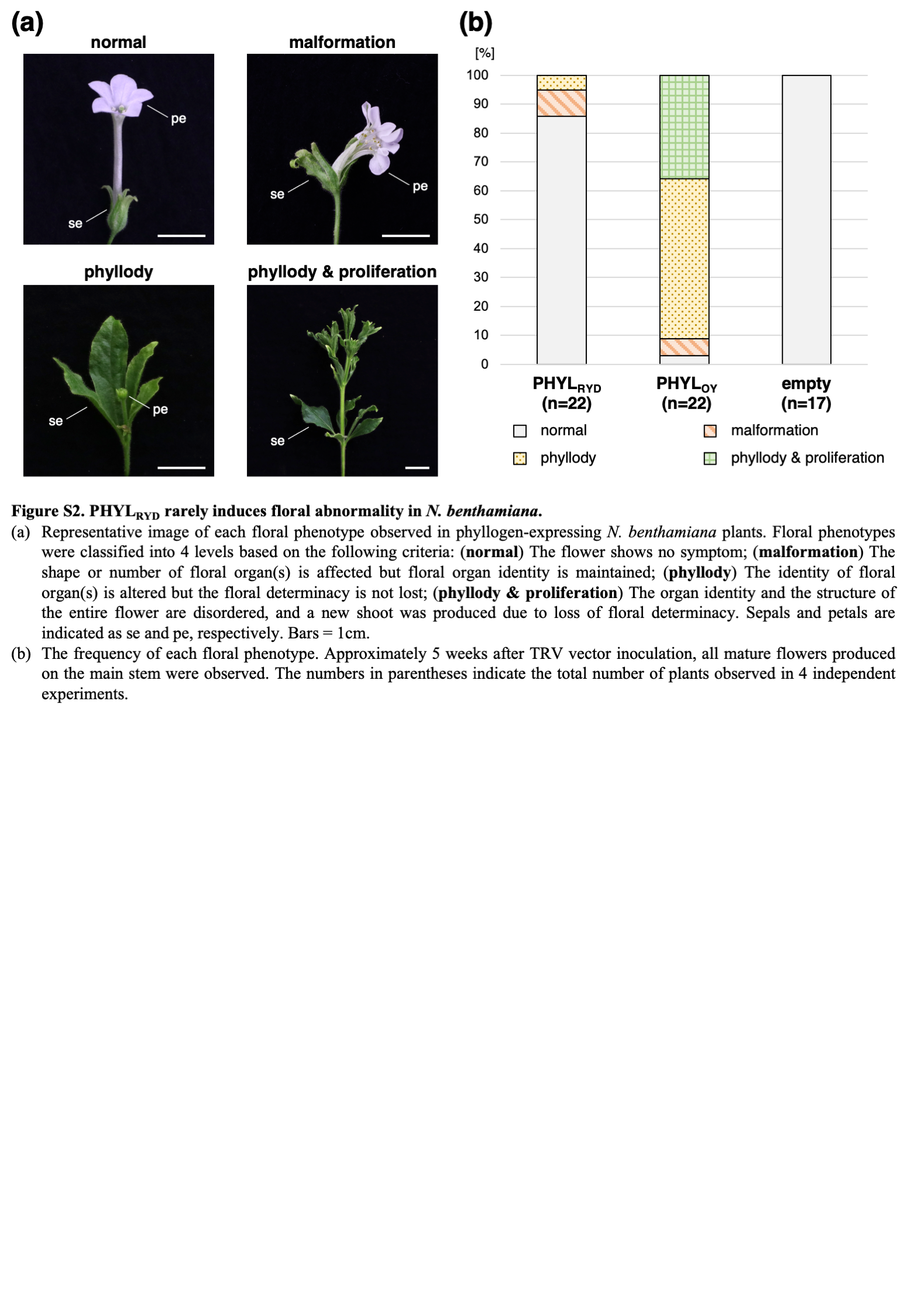

Supplement: Supplementary file 2 — Figure S2. PHYLRYD rarely induces floral abnormality in Nicotiana benthamiana. (a) Representative image of each floral phenotype observed in phyllogen‐expressing N. benthamiana plants. Floral phenotypes were classified into four levels based on the following criteria: (normal) The flower shows no symptom; (malformation) The shape or number of floral organ(s) is affected but floral organ identity is maintained; (phyllody) The identity of floral organ(s) is altered but the floral determinacy is not lost; (phyllody & proliferation) The organ identity and the structure of the entire flower are disordered, and a new shoot was produced due to loss of floral determinacy. Sepals and petals are indicated as se and pe, respectively. Bars = 1 cm. (b) The frequency of each floral phenotype. Approximately 5 weeks after TRV vector inoculation, all mature flowers produced on the main stem were observed. The numbers in parentheses indicate the total number of plants observed in four independent experiments. [file MPP-25-e13410-s004.tiff]

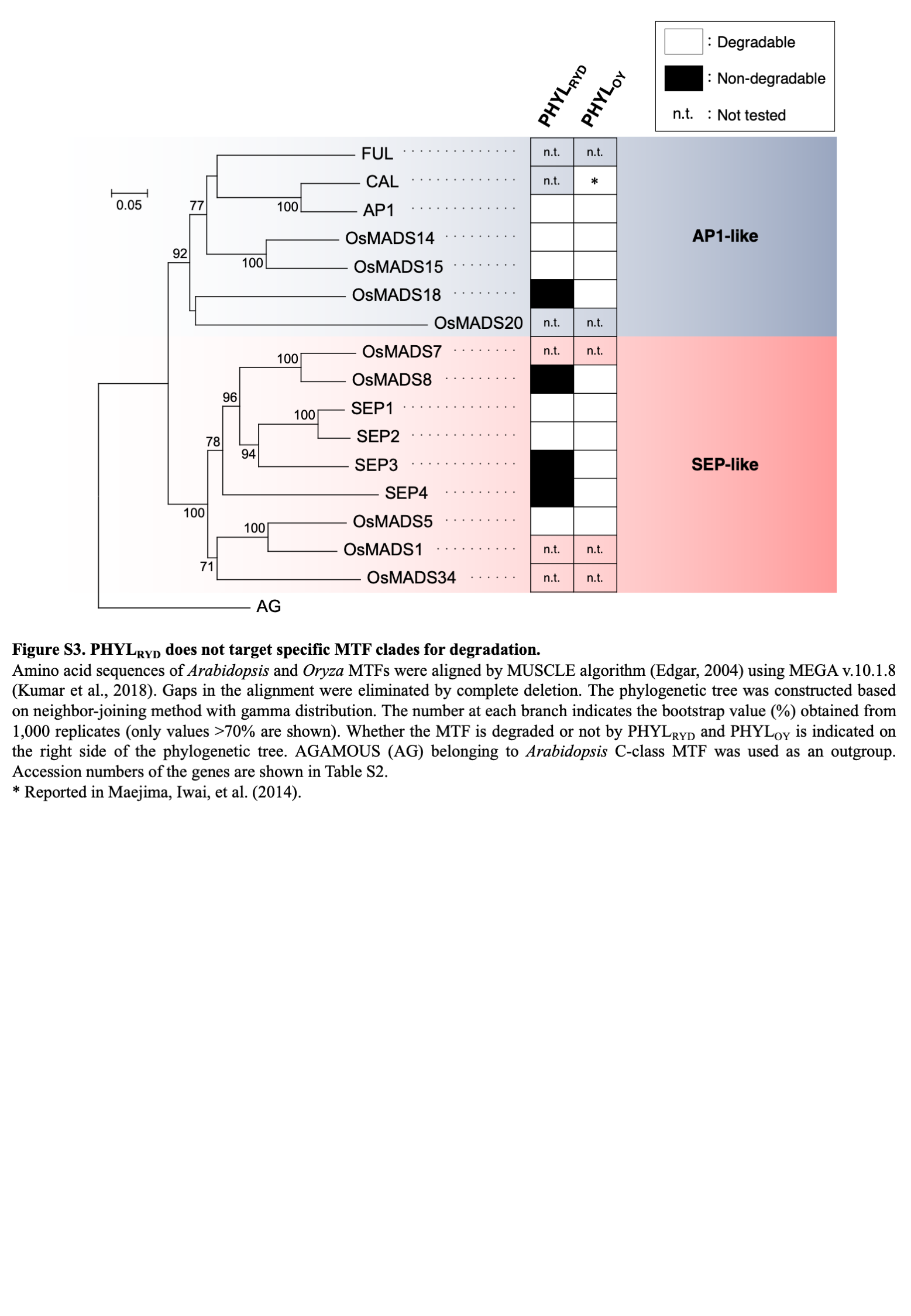

Supplement: Supplementary file 3 — Figure S3. PHYLRYD does not target specific MTF clades for degradation. Amino acid sequences of Arabidopsis and Oryza MTFs were aligned by MUSCLE algorithm (Edgar, 2004) using MEGA v. 10.1.8 (Kumar et al., 2018). Gaps in the alignment were eliminated by complete deletion. The phylogenetic tree was constructed based on neighbour‐joining method with gamma distribution. The number at each branch indicates the bootstrap value (%) obtained from 1000 replicates (only values >70% are shown). Whether the MTF is degraded or not by PHYLRYD and PHYLOY is indicated on the right side of the phylogenetic tree. AGAMOUS (AG) belonging to Arabidopsis C‐class MTF was used as an outgroup. Accession numbers of the genes are shown in Table S2. *Reported in Maejima, Iwai, et al. (2014). [file MPP-25-e13410-s007.tiff]

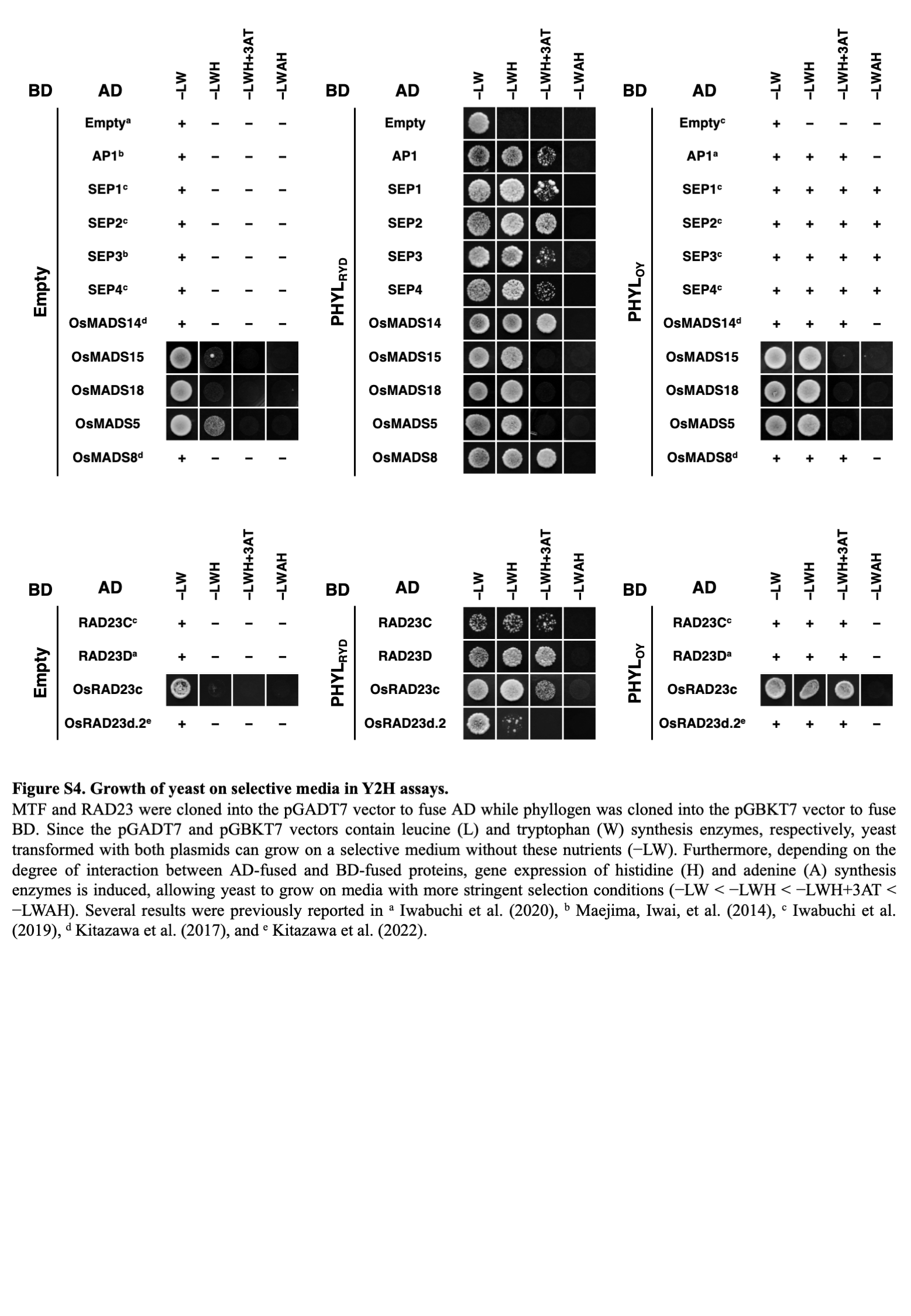

Supplement: Supplementary file 4 — Figure S4. Growth of yeast on selective media in yeast two‐hybrid assays. MTF and RAD23 were cloned into the pGADT7 vector to fuse AD while phyllogen was cloned into the pGBKT7 vector to fuse BD. Because the pGADT7 and pGBKT7 vectors contain leucine (L) and tryptophan (W) synthesis enzymes, respectively, yeast transformed with both plasmids can grow on a selective medium without these nutrients (−LW). Furthermore, depending on the degree of interaction between AD‐fused and BD‐fused proteins, gene expression of histidine (H) and adenine (A) synthesis enzymes is induced, allowing yeast to grow on media with more stringent selection conditions (−LW < −LWH < −LWH + 3AT < −LWAH). Several results were previously reported in aIwabuchi et al. (2020), bMaejima, Iwai, et al. (2014), cIwabuchi et al. (2019), dKitazawa et al. (2017), and eKitazawa et al. (2022). [file MPP-25-e13410-s008.tiff]
